# Absence of formyl peptide receptor 1 causes endometriotic lesion regression in a mouse model of surgically-induced endometriosis

**DOI:** 10.18632/oncotarget.25823

**Published:** 2018-07-31

**Authors:** Roberta Fusco, Ramona D’amico, Marika Cordaro, Enrico Gugliandolo, Rosalba Siracusa, Alessio Filippo Peritore, Rosalia Crupi, Daniela Impellizzeri, Salvatore Cuzzocrea, Rosanna Di Paola

**Affiliations:** ^1^ Department of Chemical, Biological, Pharmaceutical and Environmental Sciences, University of Messina, Messina, Italy; ^2^ Department of Pharmacological and Physiological Science, Saint Louis University, St. Louis, MO, USA

**Keywords:** endometriosis, Fpr1, mouse, inflammasome, knockout

## Abstract

Endometriosis is a female disease in which endometrial tissues grows outside the uterus. Patients showed alterations in endocrine and immune systems. Endometriotic lesions are characterized by deregulated interaction between immune cells and tissue cells. The formyl peptide receptor 1 (Fpr1) is expressed by both immune and stromal cells including epithelial cells. We investigated the development of the physiopathology of the surgically-induced endometriosis in Fpr1 KO mice compared to WT animals. Operated Fpr1 KO mice showed lower duration of uterine pain behaviors, lower size of developed cysts and reduced mast cell numbers. Immunohistochemical analyses indicated changes in NGF, VEGF and ICAM-1 expression associated with the pathology, which were reduced in absence of the Fpr1 gene. Molecular analyses indicated that in absence of Fpr1 there was reduced neutrophils accumulation and nitrosative stress formation, NF-κB translocation into the nucleus as well as NRLP3 inflammasome signalling. Fpr1 gene deletion caused reduction of resistance to the apoptosis, assessed by TUNEL assay. We underline the pathogenic role of Fpr1 in experimental endometriosis, which is the result of modulation of immune cell recruitment, suggesting it as a new target to control the pathologic features of endometriotic lesions.

## INTRODUCTION

Endometriosis is a debilitating disease which affects reproductive-age women. It is characterized by the attendance of endometrial glands and stroma outside the uterus. Often it is as a consequence of insufficient clearance of emigrant tissues following retrograde menstruation, as postulated by Sampson [1]. He supposed that during menstruation endometrial cells and stroma were regurgitated outside the fallopian tubes and then implanted into ovary and peritoneum. Nonetheless, most scientists and physicians now identify endometriosis as a systemic disease causing alterations of both the immune and endocrine systems [2, 3]. Other pathogenetic hypothesis are the endometrial steam cell implantation and Mullerian remnant abnormalities [4]. Approximately 5% of women is affected by this disease, which is associated with pelvic pain and infertility. Several evidences have underline a genetic aetiology of this pathology, in particular with 51% of hereditability [5]. There are seven risk loci associated with endometriois [6]. While the specific mechanism of the pathology is not well defined, it is established that chronic inflammation not only contributes to the development of the ectopic endometrial growth but it also promotes the development of the adhesive pathway [7, 8]. Contemporary knowledge on the endometriosis shows that development of the disease affects changes in proinflammatory environment, structural elements, apoptosis and angiogenesis [9]. The innate immune system, with macrophages and neutrophils have a key role in the induction of the endometriosis’ inflammatory process through the releasing of proangiogenic factors and inflammatory cytokines [10]. Formyl peptide receptors (FPRs) are expressed on macrophages, monocytes and neutrophils [11]. Mouse neutrophils express orthologous of human Fpr1 and Fpr2 [12, 13]. This two receptors can bind some pro-inflammatory peptides with chemotactic activity and release reactive oxygen species (ROS) and granule constituents from immune cells [14, 15]. In particular it has been described the up-regulation of the Fpr1 in patients affected by endometriosis and its role in cell differentiation and proliferation [16]. Moreover, macrophages and other immune cells express also the inflammasome, a multiprotein complex. Inflammasomes have been found also in epithelial cells of tissues and mucosal surface. Four inflammasomes are identified, of these NLR Family Pyrin Domain Containing 3 (NLRP3) is the most characterized. The activation of this inflammasome complex has been described in the physiopathology of the endometriosis [17], in association with chronic pain as well [18]. NLRP3 is a complex protein implicated in the induction of innate immune/inflammatory responses. The complex contains of the NLRP3 protein, which is a sensor for the activation of the inflammasome, and an apoptosis-associated speck-like protein containing a CARD complex (ASC), which binds pro-caspase through its CARD domain. Pro-caspase is then activated in caspase, which, in turn, catalytically cleaves pro-inflammatory cytokines (proIL-1β and pro-IL-18) to their active forms. IL-1β and IL-18 to enhance inflammation by recruiting additional inflammatory and immune cells [19]. Considering the multifactorial and complex pathogenesis of the endometriosis, in this paper, we investigate how gene deletion of mouse Fpr1 can impact on the macropscopic and microscopic features of the endometriosis, leading to an outcome related with a modulation of inflammasome signalling in the injured tissue.

## RESULTS

### Effect of absence of Fpr1 on pain behaviours

**Figure 1:**
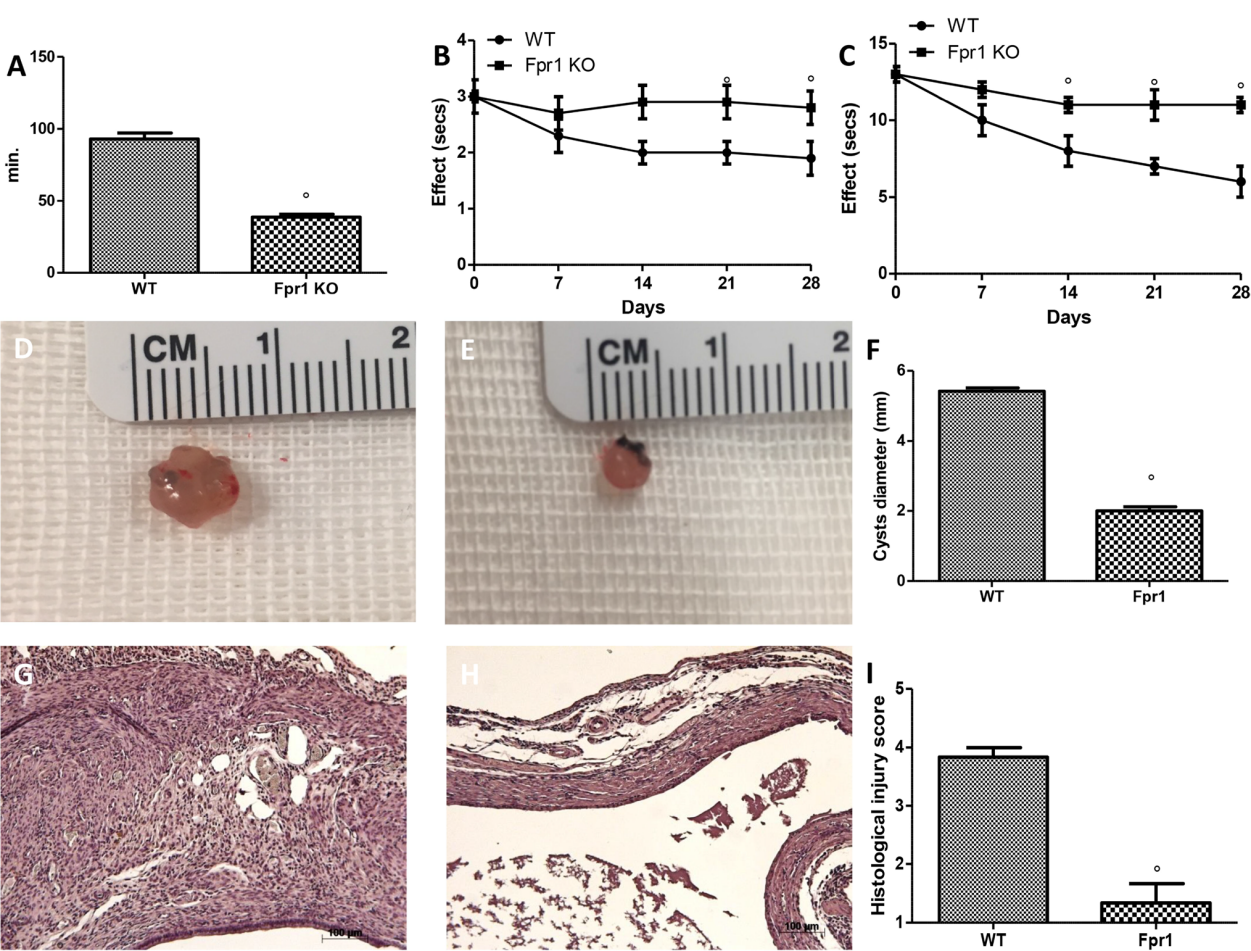
Effect of Fpr1 gene deletion on pain behaviours, cyst diameter and histological alterations Fpr1 OK mice showed shorter uterine crises than WT animals (**A**). During the tail-flick (**B**) and hot plate (**C**) tests Fpr1 OK animals showed significant less nociceptive behaviours compared to the WT mice. The absence of the Fpr1 gene significantly reduced cyst size (**E** and **F**) compared with control animals (**D** and F). Histological investigation exhibited the presence of endometrial tissue. Samples from the control group enclosed cellular infiltration and edema (**G** and **I**), and the absence of Fpr1 reduced the strictness of histological marks of endometriotic implants (**H** and I). Data are means ± SD of 10 animals for each group. ^°^*P* < 0.05 vs. WT group.

We investigated directly the effect of the Fpr1 gene deletion on pain the animals’ pain behaviours. The Fpr1 KO mice showed significantly lower duration of uterine pain behaviors than WT animals (Figure 1A). Fpr1 deletion resulted anti-nociceptive effect in tail-flick (Figure 1B) and hot plate test (Figure 1C), compared to the WT mice.

### Effects of absence of Fpr1 on the degree of endometriosis

At 28 days endometriotic implantation, both groups developed transparent cystic area in the mesentery and abdominal wall. WT (Figure 1D) and Fpr1 KO (Figure 1E) mice did not showed a different number of cyst, but cyst size was smaller in Fpr1 KO mice compared to control animals (*P* < 0.05) (Figure 1F). Histological evaluation of samples from WT animals displayed the presence of glandular endometrial epithelium and stroma, intense cellular infiltration and edema (Figure 1G and 1I). The absence of Fpr1 gene reduced the severity of the histological marks of endometriotic cysts (Figure 1H and 1I).

### Effects of absence of Fpr1 on collagen deposition, mast cell density and NGF expression

**Figure 2:**
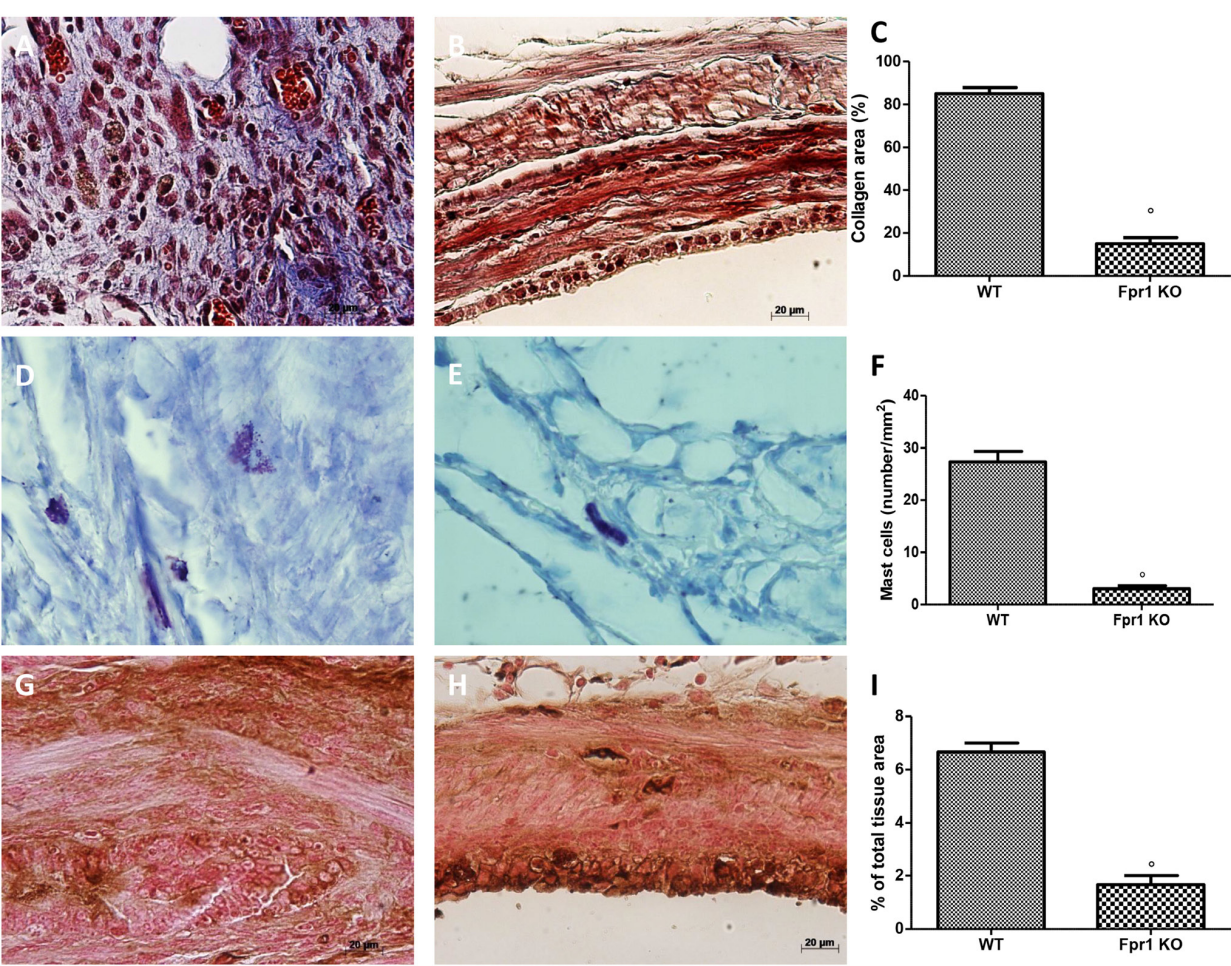
Effect of Fpr1 gene deletion on Masson trichrome staining, mast cells number and NGF expression Masson trichrome staining of endometriotic explants showed an abundant collagen deposition in the WT group (**A** and **C**), while a small amount of collagen was found in tissues from the Fpr1 KO group (**B** and C). Mast cell numbers was also lower in the Fpr1 KO animals (**E** and **F**) compared to the WT (**D** and F). Positive staining for NGF was significantly increased in WT mice (**G** and **I**), whereas in Fpr1 KO mice NGF expression was reduced (**H** and I). Data are means ± SD of 10 animals for each group. ^°^*P* < 0.05 vs. WT group.

Masson trichrome displayed distinctive staining pattern, in particular connective tissue was blue, cytoplasm stained red/pink, and nuclei stained dark red/purple. Collagen deposition (Figure 2C) was much more abundant in the WT animals (Figure 2A, and 2C) than in the Fpr1 KO group (Figure 2B and 2C). Mast cells numbers were enumerated by staining with toluidine blue (Figure 2F). Compared with the WT group (Figure 2D and 2F), mast cell numbers in the Fpr1 KO group was significantly reduced (Figure 2E and 2F). Several evidences suggest NGF triggers mast cell degranulation. Immunoistochemical analysis for NGF showed a significant increase of its staining in endometriotic tissues from WT mice (Figure 2G and 2I), whereas Fpr1 KO animals displayed a reduction of such positive staining (Figure 2H and 2I).

### Effects of the absence of Fpr1 on VEGF, ICAM, MPO expression and nitrotyrosine formation

**Figure 3:**
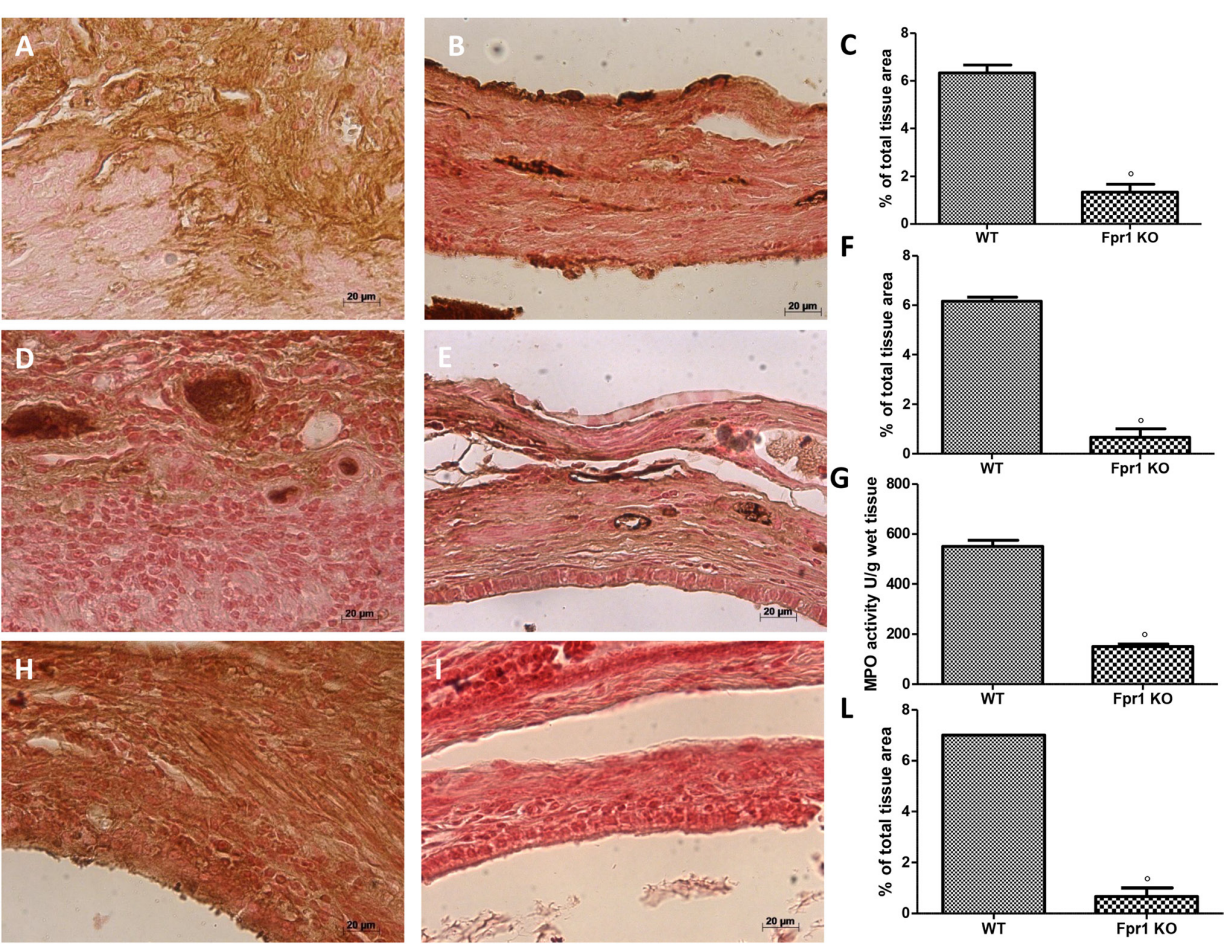
Effect of Fpr1 gene deletion on VEGF, ICAM, MPO and nitrotyrosine expression An increased expression of VEGF was found in cyst tissue from WT animals (**A** and **C**), in Fpr1 KO animals this positive staining was reduced (**B** and C). ICAM-1 expression was up-regulated in tissues from WT mice (**D** and **F**), while in Fpr1 KO animals it was lower (**E** and F). Also MPO activity was reduced in Fpr1 KO group compared to the WT group (**G**). Samples from WT animals exhibited positive immunostaining for nitrotyrosine (**H** and **L**), whereas in cyst tissues collected from Fpr1 KO group this staining was reduced (**I** and L). Data are means ± SD of 10 animals for each group. ^°^*P* < 0.05 vs. WT group.

Twenty eight days after implantation immunohistochemical analysis of the endometriotic lesions 28 displayed increased staining for VEGF in WT animals (Figure 3A and 3C), which was reduced in Fpr1 KO mice (Figure 3B and 3C). Moreover, also positive immunostaining for ICAM-1 was up-regulated in the vessels of tissues collected from WT group (Figure 3D and 3F). Tissues from Fpr1 OK animals displayed a reduction of ICAM-1 staining (Figure 3E and 3F). Explanted tissues exhibited an important accumulation of neutrophils, which was assessed by MPO activity. An increased MPO activity was found in endometriotic samples from WT mice (Figure 3G), which was reduced in Fpr1 animals (Figure 3G). To investigate the presence of nitrosative stress, immunohistochemical analysis of nitrotyrosine was performed. Endometriotic tissues from WT group showed positive nitrotyrosine staining (Figure 3H and 3L), whereas Fpr1 KO mice resulted in a substantial reduction (Figure 3I and 3L).

### Effects of the absence of Fpr1 on IκB-*α* degradation and NF*-κB* activation

**Figure 4:**
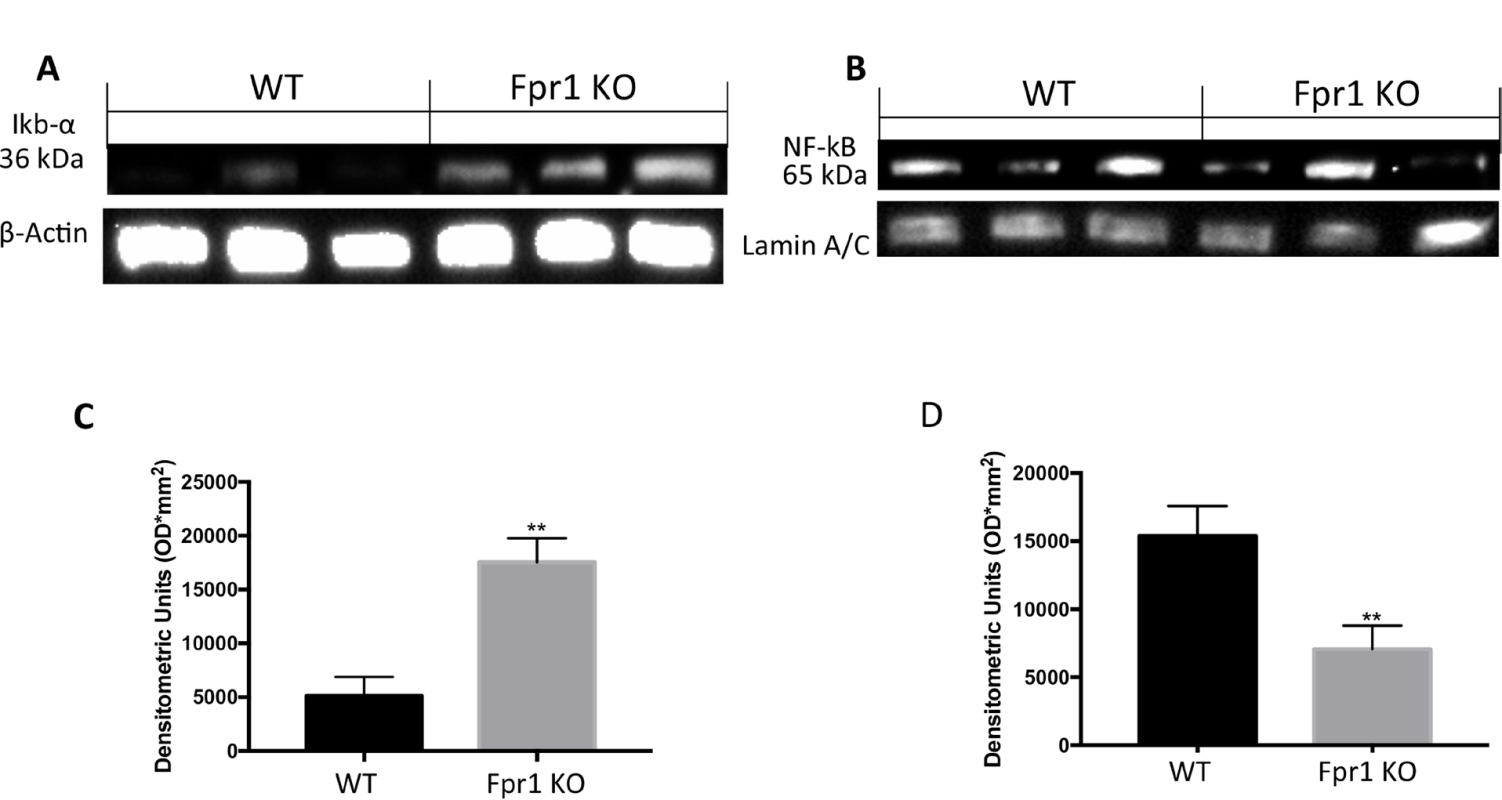
Effect of Fpr1 gene deletion on IκB-α degradation and NF-κB Tissue samples from WT animals exhibited less IκB-α expression while in Fpr1 KO mice IκB-α degradation was not allow (**A** and **C**). NF-κB levels in nuclear fractions were remarkably increased 28 days after surgery; in Fpr1 KO group these levels decreased (**B** and **D**). Data are means ± SD of 10 animals for each group. ^°^*P* < 0.05 vs. WT group.

Western Blot analysis of samples from WT mice exhibited low IκB-α expression compared to the Fpr1 KO group (Figure 4A and 4C). Conversely, NF-*κB* levels were noticeably increased in WT animals and decreased in Fpr1 KO mice 28 days after implantation (Figure 4B and 4D).

### Effects of the absence of Fpr1 on NRLP3 inflammasome pathway

**Figure 5:**
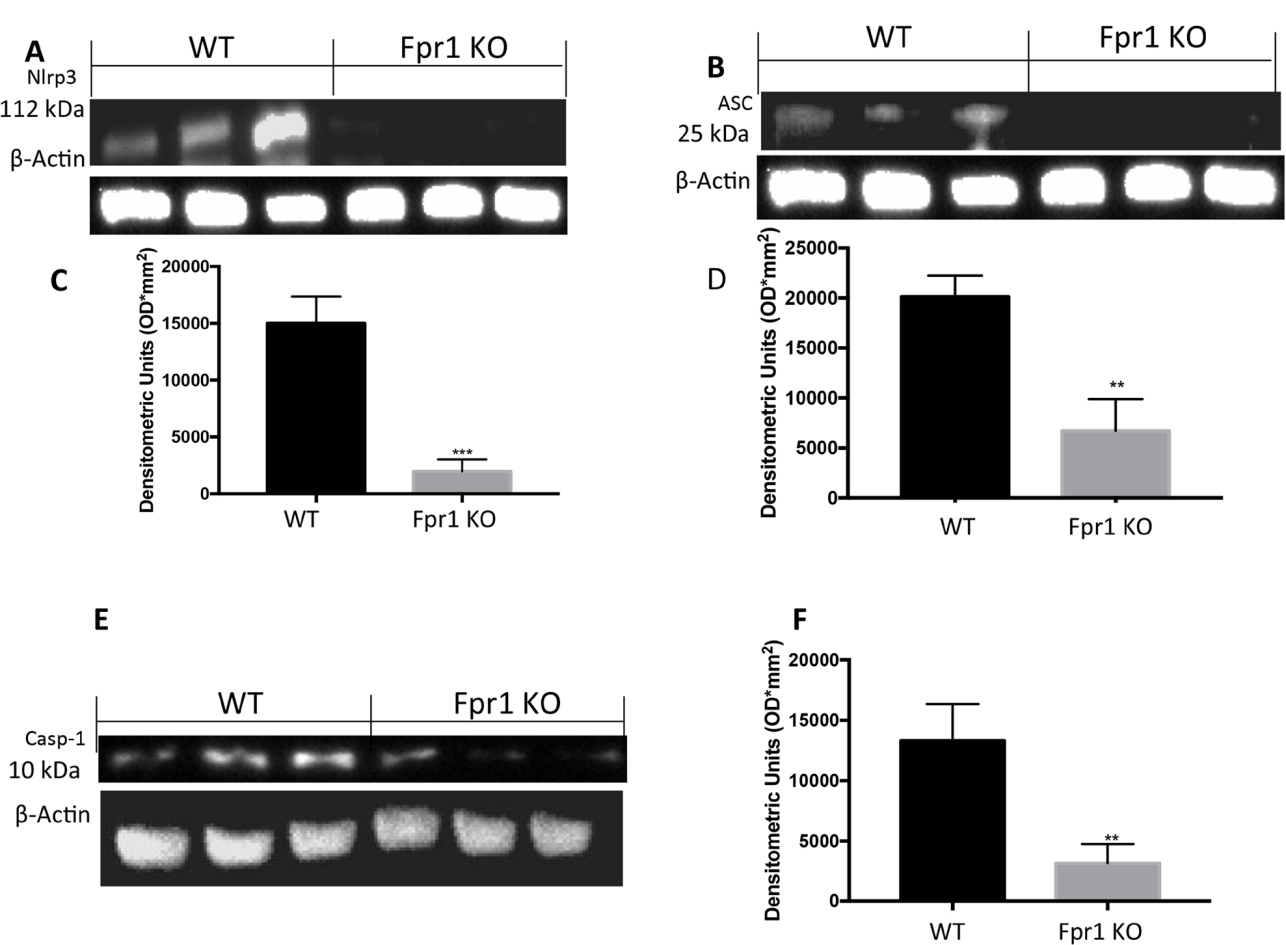
Effect of Fpr1 gene deletion on NRPL3, ASC and Caspase-1 expression Cyst tissues collected from WT animals showed positive expression of NRLP3, Fpr1 gene deletion reduced this expression (**A** and **C**). Western blot analysis displayed also increased levels of ASC in tissues from WT animals, compared to the Fpr1 KO mice (**B** and **D**). an over expression of Casp-1 was found in WT mice, while Fpr1 KO down-regulated Casp-1 expression (**E** and **F**). Data are means ± SD of 10 animals for each group. ^°^*P* < 0.05 vs. WT group.

In order to investigate the effect of the Fpr1 gene deletion on the NRLP3 inflammasome pathway we examined the NRLP3 expression. Cyst tissues collected from WT animals showed positive expression of NRLP3. In Fpr1 KO group this expression was reduced (Figure 5A and 5C). Western Blot analysis also displayed an up-regulation of ASC levels in WT group, which was reduced in Fpr1 mice (Figure 5B and 5D) confirmed this data. We found also an increased Casp-1 expression in tissues from WT group, compared to the Fpr1 KO mice (Figure 5E and 5F).

### Effects of the absence of Fpr1 on IL-1β and IL-18 expression

**Figure 6:**
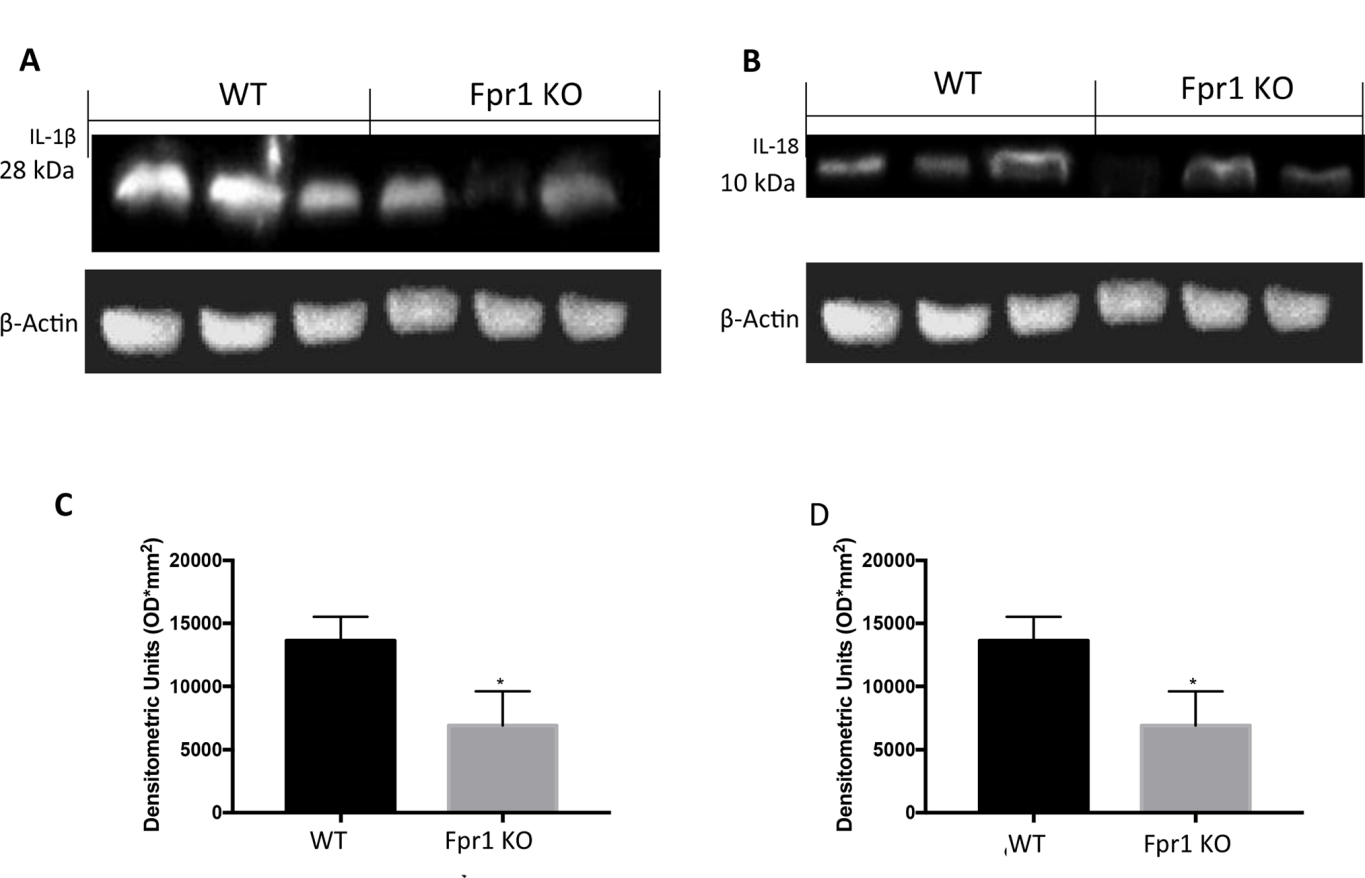
Effect of Fpr1 gene deletion on IL-1β and IL-18 expression Samples taken from WT mice exposed positive expression of IL-1β. There was a substantial reduction IL-1β expression in cyst of Fpr1 KO mice (**A** and **C**). Twenty eight days after endometriotic implants positive IL-18 expression in samples collected from WT animals was found. This expression was reduced in Fpr1 KO group (**B** and **D**). Data are means ± SD of 10 animals for each group. ^°^*P* < 0.05 vs. WT group.

Western Blot analysis revealed positive expression of IL1β and IL18 in cysts obtained from WT mice (Figure 6A and 6C; 6B and 6D respectively). In explanted tissues from Fpr1 KO mice IL1β and IL18 expression were decreased (Figure 6A and 6C; 6B and 6D respectively).

### Effects of the absence of Fpr1 on apoptosis

**Figure 7:**
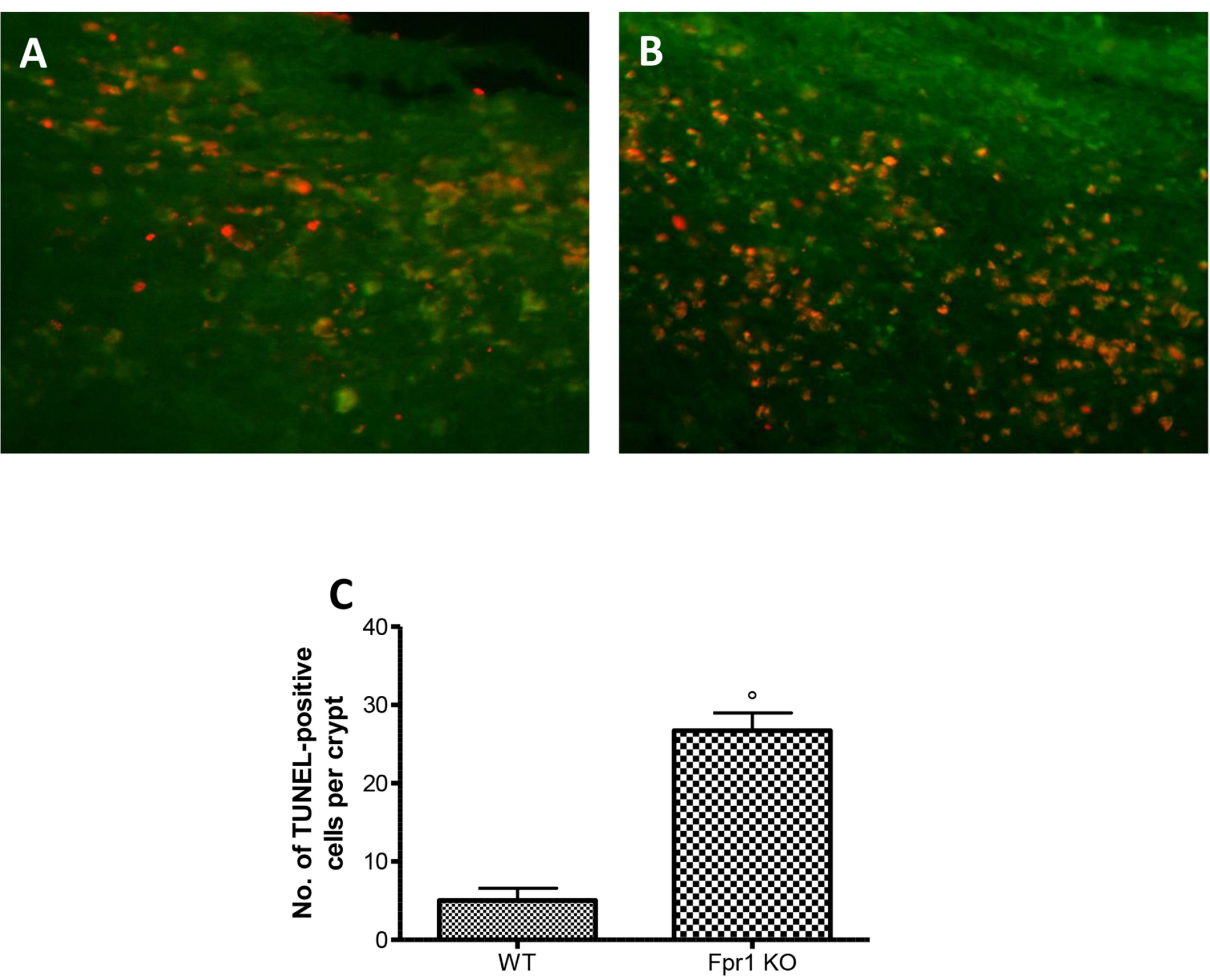
Effect of Fpr1 gene deletion on apoptosis Tunel assay of endometriotic explants from WT animals showed low co-localization (**A** and **C**), while tissues from Fpr1 KO animals revealed a important increased the cells undergoing apoptosis (**B** and C). Data are means ± SD of 10 animals for each group. ^°^*P* < 0.05 vs. WT group.

Disparity between endometriotic cell growth and apoptosis characterized endometriosis. To quantify cells undergoing apoptosis TUNEL assay was used. Tissue samples from WT animals displayed a resistance to the apoptosis (Figure 7A and 7C), while explants from Fpr1 KO mice showed a significant increase of co-localization, indicating increased apoptotic activity and decreased cellular proliferation (Figure 7B and 7C).

## DISCUSSION

The current work demonstrates that the altered Fpr1 gene expression profile allows endometriotic lesion regression in an autologous mouse model of surgically-induced endometriosis. Paula *et al.* reported morphological alteration in the endometriotic lesions from abdominal wall endometrioma associated with inflammatory response characterized by increased inflammatory cell influx [16]. In addition, the undifferentiated glandular pattern of endometriotic lesions was positively associated with an increased expression of FPR1 in the epithelial cells. The eutopic and ectopic endometrial had similar immunoreactivity to FPR1, particularly in the inflammatory, stromal and endothelial cells. Implantation, proliferation, angiogenesis, and inflammation play a central role in the initiation and growth of endometriotic lesions: in the present study we showed that the absence of Fpr1 was able not only to reduce cyst diameter and histopathological score of endometriosis but also to ameliorate the suffering induced by the disease. In this pathology collagen deposition is an important index of the adhesions developing in mice [20]. The invasion and adherence of endometriotic cells require an increased production of collagen [21], animals with Fpr1 gene deletion showed minimal collagen deposition compared to the WT. The development of implants is associated with an increased number of intact and degranulated mast cells [22–24]. They play an important function in the induction of the disease, the adhesion process and the production of pelvic pain [25, 26]. Chronic pain associated with endometriosis is one the most frequent motive for medical consultation [27]. A lot of data suggest that activated mast cells are placed close to the peripheral nerves [28] to release molecules, such as NGF, which may contribute to the chronic pain [29]. Fpr1 KO mice displayed a reduction of mast cells number and also a significant decrease in NGF expression in the explanted tissues. This result well correlates with the reported reduction of the animal’s suffering. Mast cells are important also for neo-angiogenesis [30], which guarantees oxygen supply to implants. The reduction of mast cells number caused by Fpr1 gene deletion led also to a down-regulation of VEGF, which is normally released by mast cells during inflammation [31]. Probably the reduced neo-angiogenesis is responsible for the smaller cyst diameter found in Fpr1 KO mice. Another important mediator involved in the pathological development of the endometriosis is ICAM-1 [32]. It mediates the interaction between endothelial cells and neutrophils at the adhesion phase [26]. Animals with deletion of Fpr1 gene showed a reduction of the positive staining for ICAM-1 compared to the WT mice. This data is well in line with the decreased leukocyte infiltration found in endometriotic implans [33]. Fpr1 is highly expressed on neutrophils and promotes their migration into the injured tissues [11]. Additionally, mitochondria derived N-formylated peptides are powerful agonist for Fpr1 and controls cell migration following host cell damage. In order to verify the effect of the Fpr1 gene KO on myeloid cell types MPO activity was evaluated. In WT animal was found a significant cytoplasmic reactivity for MPO, which was decreased in Fpr1 KO animals. The development of the endometriosis leads also to an over-expression of reactive oxygen species (ROS), such as superoxide anion and peroxynitrite. Their reactions cause lipid peroxidation, oxidization of sulfhydryl groups and nitration of tyrosine residues. Tissues from Fpr1 KO animals showed reduced staining for nitrotyrosine [15]. Frp1 activation allows guanosine diphosphate (GDP) conversion in guanosine triphosphate (GTP). It activates phospholipase C *β* (PLC*β*) [34], which in turn hydrolysis 4,5-bisphosphate (PIP2) into inositol 1,4,5-trisphosphate (IP3). IP3 releases calcium from endoplasmatic reticulum, activating the calmodulin (CaM)/calcineurin pathway and DAG. DAG activates PKC isoforms, which are responsible of the NF-κB translocation to the nucleus. The protective effects of the Fpr1 gene deletion may depend, in part, from the down-regulation of the NF-kB pathway. NF-kB plays a key role in the immune and inflammatory response and modulates cell proliferation, apoptosis, adhesion, invasion, and angiogenesis in many cell types. Tissues from Fpr1 KO mice exhibited a reduced IkB*α* degradation and NF-*κ*B translocation to the nucleus induced by endometriosis. This reduced releasing of NF-*κ*B and migration to the nucleus caused less activation of the transcription of target genes involved in the inflammatory process, such as the inflammasome. Damaged cell mediators released by endometrial cell and an inappropriate immune response are also able to activate the inflammasome pathway. It is a multiprotein complex often attributed to macrophages, but it has also been found in mucosal tissues and epithelial cells [17]. The enhanced expression of NRLP3 in the inflammatory lesions has been reported [35]. Fpr1 gene deletion led to a reduced activation of all the members of the NRLP3 inflammatory complex. In particular NLRP3 has an ATPase activity, which is necessary to oligomerize ASC and activate Caspase-1 [36]. The activated form of Active Caspase-1 in turn cleaves pro-IL-1*β* and pro-IL-18 into them active forms [37, 38]. Probably the reduced expression of all this mediators was due to the down-regulation of the NF-*κ*B pathway mediated by the absence of Fpr1. The endometriotic process is also associated with the resistance to apoptosis [39]. We identified showed increasing apoptosis measured by TUNEL assay associated with the Fpr1 KO gene. Collectively, the data shows that animals with a deletion of Fpr1 gene displayed reduced inflammation and pain induced by an experimental mouse model of endometriosis, suggesting it as a new target to control the pathologic features of endometriotic lesions.

## MATERIALS AND METHODS

### Animals

Formyl peptide receptor 1 gene-deficient mice on the C57BL/6 genetic background and C57BL/6 mice, that were used as wild type controls, were acquired from Envigo (Milan, Italy) and located in controlled cages with standard rodent water and chow. Cages were placed in room kept at 22 ± 1° C with a 12h light and dark cycle.

### Ethical statement

The Review Board for the care of animals of the University of Messina approved the study (Protocol number 8/U-apr16). All animal experiments obeyed with regulations in USA (Animal Welfare Assurance No A5594-01, Department of Health and Human Services, USA), Europe (O.J. of E.C. L 358/1 12/18/ 1986), Italy (D.M. 116192) and the ARRIVE guidelines.

### Induction of experimental endometriosis

Endometriosis was performed using homologous uterine horn transplantation. The animal were anesthetized with Isoflurane laparotomy was performed, the left uterine horn was exposed and excised (Pelch *et al.*, 2010). This piece of tissue was located in phosphate-buffered saline (PBS) 37° C. Subsequently it was cut along the longitudinal axis to have two fragments of 5 × 5 mm. One of the two cubes was sutured into the abdominal wall, with the endometrial layer fronting a large vessel, and the other one to the mesentery nearby to a large vessel. Sterile 6-0 silk suture was used to suture the implants while and the abdominal cavity was locked using 4-0 silk suture. For five days after the surgery penicillin was administered (40,000 U/kg).

### Experimental groups

Mice were randomly divided in following groups (*n* = 20 for each):

WT group: WT mice were subjected to experimental endometriosis as described above;

Fpr1 KO group: mice were subjected to experimental endometriosis as described above, as well as WT group.

The minimum number of animals for each group was calculated using the statistical test a priori power analyzes of the G-power software. This statistical test provides an efficient method for determining the sample size necessary to perform the experiment before the experiment the same is actually conducted.

Randomization was based on a single sequence of random assignments. This technique led to a complete randomness of the transfer of a subject to a specific group. We used a simple randomization method: flipping a coin. For our two groups (WT versus Fpr1 KO), the side of the coin (i.e., heads - WT, rood- Fpr1 KO) decided the assignment of each subject.

Mice were sacrificed at twenty-eight days after endometriosis induction. Animals were anaesthetized with isoflurane; laparotomy was executed to collect the implants and perform all the histology and biochemical studies.

### Quantification of pain behaviours

#### Uterine pain behaviours

Uterine pain behaviours were measured looking at 4 positions: “lambda” position (the mouse’s back formed a triangular shape angle relative to the floor), “alpha” position (the mouse had the abdomen adherent to the floor while nose curving towards the tail), “stretch-flat” position (stretching of the abdomen adherent to the floor), and “squash-pelvic” position (in a standing or sitting position pressing the abdomen to the floor). For each mouse the uterine pain behaviours were calculated as the sum of duration of all position and reported as global duration of uterine positions.

#### The tail-fick method

Mouse tail was located in 50 ± 0.5° C warm water and the time between input and withdrawal from the water was measured (3 tests were performed). The latency was reported with a sensitivity of 0.01 sec. A maximum tail-flick inactivity of 10 sec. was employed to minimise damage to the tail.

#### The hot-plate method

The hot-plate latency was investigated by placing the mouse on a metal surface maintained at 53.6° C (Hot Plate, Ugo Basile, Milan, Italy). The animal was observed during the measurements. Time between input and the licking of a hind paw was recorded. Maximal latency used was 45 sec.

### Histology

Endometriotic implants were collected 28 days post-surgery. Tissues were fixed at room temperature in buffered formaldehyde solution (10% in PBS) for 24 h, then dehydrated through a graded series of ethanol, embedded in Paraplast (Sherwood Medical, Mahwah, NJ) and cut into 7-mm-thick sections. Sections were deparaffinized with xylene, stained with hematoxylin/eosin (H&E) and analysed using a Axiovision Zeiss (Milan, Italy) microscope. Damage was estimated by an expert histopathologist blinded to the study, and scored as follows: 0 = no implantation, 1 = cellular infiltration, 2 = edema, 3 = continuous inflammatory lesions, 4 = presence of glandular epithelium and stroma.

### Masson trichrome and toluidine blue staining

Explanted sample sections were stained with Masson trichrome according to the manufacturer’s protocol (Bio-Optica, Italy, Milan). Tissue slides were stained also with toluidine blue. Samples were deparaffinized in xylene and dehydrated using a graded series of ethanol. Then, sections were placed in water for 5 min and transferred to toluidine blue for 4 min, blotted carefully. Sections were embebbed in absolute alcohol and subsequently cleared in xylene, mounted on a glass slide using Eukitt (Bio-Optica, Italy, Milan). Metachromatically stained mast cells were enumerated by counting 5 high-power fields (40×) per section using Axiovision Zeiss (Milan, Italy) microscope.

### Immunohistochemical localization of nitrotyrosine, VEGF, nerve growth factor (NGF) and intercellular adhesion molecule (ICAM)

At 28 days from surgery, endometriotic implants were fixed in 10% (w/v) PBS-buffered formaldehyde and embedded in paraffin. Seven µm sections were prepared from tissues. After deparaffinization, endogenous peroxidase was quenched with 0.3% (v/v) hydrogen peroxide in 60% (v/v) water for 30 min. The slides were permeabilized with 0.1% (w/v) Triton X-100 in PBS for 20 min. Tissue sections were incubated in 2% (v/v) normal goat serum in PBS to block non-specific binding. Sequential incubation for 15 min with avidin and biotin (Vector Laboratories, Burlingame, CA) was performed to block, respectively, endogenous avidin or biotin binding sites. Sections were then incubated overnight with: anti-nitrotyrosine antibody (Millipore), anti-VEGF antibody (A-20: sc-152, Santa Cruz Biotechnology), anti-NGF antibody (E-12: sc-365944 Santa Cruz Biotechnology) or anti-ICAM antibody (G-5: sc-8439, Santa Cruz Biotechnology). Samples were washed with PBS, and incubated with secondary antibody. Specific labeling was identified with a biotin-conjugated goat anti-rabbit IgG and avidin–biotin peroxidase complex (Vector Laboratories, Burlingame, CA). Immunohistochemical images were collected using a Zeiss microscope and Axio Vision software. For graphic display of densitometric analyses, the intensity of positive staining (brown staining) was measured by computer-assisted color image analysis (Leica QWin V3, UK). The percentage area of immunoreactivity (determined by the number of positive pixels) was expressed as percent of total tissue area (red staining).

### Myeloperoxidase activity

Myeloperoxidase (MPO) activity was quantified as previously described [40]. Twenty-eight days after surgery endometriotic implants were collected. Sample were homogenized in a solution containing 0.5% hexa-decyl-trimethylammonium bromide dissolved in potassium phosphate buffer 10 mM (pH 7) and centrifuged at 20,000 g at 4° C for 30 min. An aliquot of the supernatant reacted with a solution of tetra-methylbenzidine (1.6 mM) and 0.1 mM H2O2. Spectrophotometrically at 650 nm was measured the rate of change in absorbance. MPO activity was quantified in U per gram weight of wet tissue and was calculated as the quantity of enzyme degrading 1 µmol of peroxide min-1 at 37° C.

### Western blot analysis for NRLP3, ASC, Casp-1, IL-1β, IL-18, IkB-α and nuclear factor-kB (NF-kB)

Explants were suspended in buffer A (0.2 mM phenylmethylsulfonyl fluoride, 0.15 µM pepstatin A, 20 µM leupeptin, 1 mM sodium orthovanadate) homogenized for 2 min, and centrifuged at 10000g for 10 min at 4° C. Supernatants represented the cytosolic fraction. The pellets, containing nuclei, were re-suspended in buffer B (150 mM NaCl, 1% Triton X-100, 1 mM EGTA, 1 mM EDTA, 10 mM Tris–HCl pH 7.4, 0.2 mM phenylmethylsulfonyl fluoride, 20 µM leupeptin, 0.2 mM sodium orthovanadate). After centrifugation for 30 min at 15,000 g at 4° C, the supernatants contained nuclear proteins. Samples were stored at –80° C for further analysis. The levels of IkB-α, NRLP3, ASC and Casp1 were quantified in the cytosolic fraction, while the nuclear fraction was used to quantify NF-kB p65 levels. Membranes were blocked with 1× PBS, 5% (w/v) non-fat dried milk for 40 min at room temperature and later probed with one of the following primary antibodies: IkB-α (Santa Cruz Biotechnology), anti-NRLP3 (Santa Cruz Biotechnology, sc-66846), or anti-ASC antibody (Santa Cruz Biotechnology, N-15: sc-22514-R) or anti-Caspase**-**1 p20 (Santa Cruz Biotechnology, G-19: sc-1597) or anti-IL-1β (Santa Cruz Biotechnology) or anti-IL-18 (Santa Cruz Biotechnology) or anti-NF-kB p65 (1:400; Santa Cruz Biotechnology) in 1× PBS, 5% (w/v) non-fat dried milk, 0.1% Tween-20 at 4° C overnight. Filters were incubated with peroxidase-conjugated bovine anti-mouse IgG secondary antibody or peroxidase-conjugated goat anti-rabbit IgG (1:5000, Jackson ImmunoResearch, West Grove, PA) for 1 h at room temperature. To evaluate that blots contained equal amounts of protein they were also incubated with an antibody against β-actin (1:500, Sigma-Aldrich). Relative expression of protein bands for IkB-α (37 kDa), NF-kB p65 (65 kDa), NRLP3 (106kDa), ASC (24 kDa), Caspase-1 (20 kDa) was quantified by densitometric scanning of the X-ray films with GS-700 Imaging Densitometer (GS-700, Bio-Rad Laboratories, Milan, Italy) and a Image Lab 3.0 software (Bio-Rad, USA), and standardized to β-actin levels.

### TUNEL staining

TUNEL staining protocol was according to a Roche protocol. Sections were dewaxed in xylene and hydrated in ethanol series to water, permeabilized with citrate buffer 0.1 M and incubated in TUNEL reaction for 60 min at 37° C in the dark. Samples were rinsed in PBS 3 and then observed using an excitation wavelength in the range of 520–560 nm (maximum 540; green) and in the range of 570–620 nm (maximum 580 nm; red).

### Materials

All compounds were acquired from Sigma-Aldrich (Milan, Italy). All chemicals were of the highest commercial grade available. All stock solutions were prepared in non-pyrogenic saline (0.9% NaCl; Baxter, Italy, UK).

### Statistical evaluation

All values are expressed as mean ± standard error of the mean (S.E.M.) of N observations. For *in vivo* studies N represents the number of animals used. For histological experiments, the figures shown are representative of at least three experiments (histological staining) performed on different days on tissue sections collected from all animals in each group. The results were analyzed by 2-way ANOVA. A *P*-value of less than 0.05 was considered significant.

## Abbreviations

FPRs: Formyl peptide receptors
H&E: Hematoxylin/eosin
HCl: Hydrochloric acid
ICAM: Intracellular adhesion molecule
NLRP3: NLR Family Pyrin Domain Containing 3
MPO: myeloperoxidase
NGF: nerve growth factor
PAR: (poly-ADP)ribose polymerase activation
PLD: polydatin
ROS: reactive oxygen species
NaCl: Sodium Chloride
